# Paternalistic Leadership, Polychronicity, and Life Satisfaction of Nurses: The Role of Work-Family Conflict and Family-Work Conflict in Public Sector Hospitals

**DOI:** 10.3389/fpsyg.2021.648332

**Published:** 2021-08-20

**Authors:** Nida Gull, Zhejie Song, Rui Shi, Muhammad Asghar, Muhammad Asim Rafique, Yupeng Liu

**Affiliations:** ^1^School of Management Sciences and Engineering, Yanshan University, Qinhuangdao, China; ^2^Emergency Department, Qinhuangdao First Hospital, Qinhuangdao, China

**Keywords:** paternalistic leadership, polychronicity, work-family conflict, family to work conflict, life satisfaction, health-care staff

## Abstract

Based on the leadership literature, this study investigates how paternalistic leadership (PL) and polychronicity (PC) affect the life satisfaction (LS) of nurses, specifically in public hospitals. Moreover, the mediating role of work-family conflict (WFC) and family-work conflict (FWC) is also assessed the relationships among PL, PC, and LS. The cross-sectional study design is used in this study due to its cost benefits and the convenience of data collection at a single point in time. A survey questionnaire is used to collect data from 226 nurses, and the Partial least squares structural equation modeling (PLS-SEM) technique is used to investigate the proposed model. The findings of this study confirmed that PL and PC have a positive relationship with LS. Furthermore, WFC partially mediated the relationships among PL, PC, and LS. In addition, FWC partially mediated the relationship between PL and LS; the role of FWC in mediating the relationship between PC and LS has been found to be insignificant. Employees with high PC and those whose supervisors show PL behavior become more satisfied with their lives and have relatively low WFC and FWC. In addition, the theoretical and practical implications have also been discussed.

## Introduction

Paternalistic leadership (PL) has progressively attracted organizational researchers worldwide during the last two decades (Aycan, 2008, 2015; Aycan et al., 2013; Mittal and Bienstock, 2020). Most studies have shown that leaders generally use enticements and provide social support that impacts the life satisfaction (LS) of employees (Ampofo et al., 2017). Several studies have focused on the effect of transactional, transformational, charismatic, and ethical leadership on LS (Zhou and Miao, 2014; Jiang et al., 2017; Vem et al., 2017); however, a few studies have examined the association between paternal leadership (PL) and LS in a cross-cultural environment (Luu and Djurkovic, 2019). At present, PL behavior is urged to pay more attention to effective communication with the changing economic environment to motivate the employees (Huxtable-Thomas and Hannon, 2018). According to previous studies on leadership styles, the PL style of leadership is particularly related to the social support of employees. The relationship of PL with various outcomes has been tested in several studies (Le and Lei, 2019; Luu and Djurkovic, 2019; Al-Ghazali, 2020). After comprehensively reviewing the prior literature, a significant gap comes into sight (Conte et al., 2019; Weintraub et al., 2019) that urged us to investigate the relationship of PL and PC with life LS which is relatively less studied. Furthermore, researchers have given high importance to the PL (Mittal and Bienstock, 2019, 2020) and polychronicity (PC; Korabik et al., 2017; Mittal and Bienstock, 2019), particularly in the healthcare sector and this study contemporaneously seeks out to answer these queries: (a) Do PL and PC affect the LS of healthcare workers? (b) How and up to what level do the WFC and FWC mediate the relationships among PL, PC, and LS?

Therefore, this study investigates the direct relationship of PL and PC with LS based on the conversation of resource (COR) theory; in addition to this, we also explored the mediating roles of WFC and FWC, especially in the healthcare sector. PL is defined as a fatherly leadership style: “the combination of authority and discipline based on benevolence and morality” (Luu and Djurkovic, 2019; Mert and Ozgenel, 2020). PC describes the preference of an individual to do several activities simultaneously (Bluedorn et al., 1999). However, PC refers to “employees perform multiple tasks simultaneously” (Mittal and Bienstock, 2019). Employees under supportive and fatherly supervision cope with the WFC and FWC and become satisfied with their lives (Shih et al., 2010; Mittal and Bienstock, 2019). LS is described by Shin and Johnson (1978) as “the global assessment of a person's quality of life satisfaction according to his/her chosen criteria” (p. 478). Moreover, work-family conflicts (WFCs) and family-work conflicts (FWCs) are broad terms which refer to the degree to which work and family roles are incompatible (Aycan, 2008). The constructs are primarily based on theory and the scarcity of resources, which postulates that the demands of one role exhaust personal resources, such as time and physical or mental energy, leaving inadequate resources to allocate activities of other roles (Asghar et al., 2018). This study addresses another construct; WFC is defined as a form of conflict while performing roles; and conflict occurs when someone interrupts multiple functions (Yucel and Latshaw, 2020) and becomes dissatisfied with their lives. There are distinctive issues observed and associated with uncongenial working hours where there is a burden of work examined by Shockley et al. (2017).

The study is conducted on healthcare workers, especially on nurses of public sector hospitals. They play a vital role in hospitals because they are regarded as the backbone of the healthcare sector. Primarily, nurses work late hours, almost from 12 to 16 h (Grzywacz and Bass, 2003; Asghar et al., 2018); this routine muddles their families and creates stress, leading to frustration in their lives (Cacique et al., 2019). In addition, nurses working in the hospitals are caught in WFC while balancing their work requirements in two ways, i.e., family/domestic and outside the home/social commitment (Michel et al., 2011; Öge et al., 2018). After the detailed analysis of psychologists and neuroscientists, Cheng et al. (2014) recommended that multitasking people have a trait to switch rapidly on a series of tasks instead of working on them at once (Kononova et al., 2017). Therefore, when a multitasking employee copes with WFC and FWC, it enhances the LS of healthcare workers, urging us to conduct this study.

The contribution of this study is 2-fold; theoretically, the direct relationship between PL and PC with LS was not explored in previous studies that are thoroughly investigated in this study, especially in the healthcare sector. Moreover, the mediation role of WFC and FWC has not yet been explored between the constructs mentioned above. Therefore, a second major contribution of this study is to explore the multiple mediations of WFC and FWC between the relationship of PL and PC with LS of nurses. We considered that the healthcare sector is a highly stressful work setting for the employees compared with other services organization, but polychronic employees can better perform in such a stressful working environment. So, PL can play a crucial role in training employees showing polychronic behavior (Mittal and Bienstock, 2019, 2020).

## Literature Review and Hypothesis

Researchers have shown that substantive attention toward the leadership practices that increase job satisfaction (Shi et al., 2020) critically influences the late working hours and inequalities (Alrawadieh et al., 2020); all of these factors intensify the disturbance of quality of mental health, disturb the work-family balance (Mittal and Bienstock, 2020), and affect the well-being of employees. Thus, the PL attribute reflects that the leader must be expressive; he/she should show the benevolence of a father, dignity, and morally unselfishness (Zorlu, 2019; Khan and Gul, 2020). According to the study by Wang et al. (2020), a study on fatherly leadership is flourishing very fast. According to the study by Chen et al. (2014), the three integrated but contradictory dimensions of PL are mentioned as “benevolent leadership style, authoritarian leadership style, and finally, the moral leadership style.” PL significantly affected the perceived quality of life in several countries (Aycan et al., 2013; Cheng et al., 2014). It is argued that when the quality of life enhances, the LS level rises (Aycan, 2015; Bedi, 2020).

Both men and women are continually involved in developing their careers and carrying their families with their job (Kai, 2013; Jiang et al., 2017). For example, in hospitals, nurses find several difficulties to manage a highly demanding career by handling more than one task; along with this, they have to maintain their family setup (Lau et al., 2020). Therefore, they need the support of leaders to tackle such difficulties and work passionately along with handling their family problems. Öge et al. (2018) found paternalism (fatherly leadership style) is an antecedent to resolve work-family- and family-work-related conflicts. This concept is further supported by Ugurluoglu et al. (2018) that PL is a fundamental perception that provided the available resources to employees. Therefore, they can use them to cope with conflicts that produced favorable outcomes (Luu and Djurkovic, 2019).

In most of the hospitals where nurses work dedicatedly with heavy workloads due to long working hours, their WFC and FWC increased (Kim et al., 2005; Boyar et al., 2008). Due to excessive workload, conflicts occurred among nurses and lack of free time may cause adverse effects that ultimately make them unable to cope with these conflicts. The concept of WFC is presumed as the work and family roles are characterized as devoting effort how much they can invest energy and time for performing one role (Hungerford and Cleary, 2020), feeling burdened to perform sufficiently and effectively the other role, then someone feeling caught while performing roles and unable to fulfill their requirements (Luu and Djurkovic, 2019). The concept of Netemeyer et al. (1996) fostered an understanding of the work role, that is, when a person engages to perform work roles, his family role affects, and WFC. Many studies have been found that PL behavior is most beneficial for nurses (Farh et al., 2006; Aycan, 2008; Aycan et al., 2013), where close relationships of working nurses and their concern for the lives in and out of work can create a more friendly environment at the workplace.

Hence, the nurse employees had to dispense with first-class services to the hospital and take care of patients because they intermingle with patients in person/one on one (Asghar et al., 2020). In case of an emergency condition like a pandemic, or critical condition of patients, nurses must ensure 24 × 7 availability in the hospital (Buchan et al., 2017). Leaders of nurses should show extreme care and affection for the survival of their subordinate nurses; these leaders should also be keen and mindful of their family problems (Farh and Cheng, 2000; Kai, 2013; Bedi, 2020). Work and family are inseparable domains, so further research attention is converted to these concepts (Kubicek and Tement, 2016). The most of severe consequences result from conflicts between an individual's job and family responsibilities (Hofmann et al., 2014), the positive relationship between families LS (Jiang et al., 2017), (Anafarta and Irmak, 2009; Ryan and Sagas, 2009; Burke and El-Kot, 2010), and organizational performance (Shih et al., 2010). According to the study by Wang et al. (2017) and Conte et al. (2019), there is a direct effect of leadership on the relationship between job characteristics and WFC. This relationship declared that where a supportive leadership style exists, nurses can shape their perception while doing their jobs (Pellegrini and Scandura, 2006).

After the comprehensive literature review, it is discovered that PL was focused on how it impacts the two domains of WFC and FWC (Sanz-Vergel et al., 2015; Mittal and Bienstock, 2020). However, many scholars have argued that PL affects the FWC and WFC (Jiang et al., 2017; Öge et al., 2018). By taking this concept, the positive linkage of PL leadership style with the behavior of employees toward job attitudes, performance, job satisfaction, and organizational citizenship behavior has been shown (Chen, 2017; Teng and Chen, 2019). This means the knowledge gaps in the relationship of PL, WFC, FWC, and LS, which can be adequately addressed (Kaya et al., 2020). Through the lens of these concepts, it is suggested and put forward to analyze empirically whether any positive relationship between fatherly behavior of PL and LS of employees of those nurses working under paternalistic leaders.

- *P1. The fatherly behavior of the paternalistic leader is positively related to the LS of the employee*.
- *P2. The fatherly behavior of the paternalistic leader is negatively linked with WFC*.
- *P3. The fatherly behavior of the paternalistic leader is negatively linked with FWC*.
- *P4. WFC mediates the relationship between the paternalistic leader and the LS of the employee*.
- *P5. FWC mediates the relationship between the paternalistic leader and the LS of the employee*.

The shortage of nurses has become a burning issue in Pakistan because of its adverse effects on the work life of nurses and personal life (Haupt and Ebner, 2020). Due to the shortage of nursing staff, they have to cope with several tasks at a time and manage the work burden. By taking this concept, the term PC is introduced as it is the seminal work by Bluedorn et al. (1992) and Conte et al. (2019), from where this term has evolved. PC at the individual level has been defined as the multitasking behavior (Konig et al., 2005; Kononova et al., 2017), preference of a person to perform tasks “more than one at once” (Sehrish and Zubair, 2020). For employees who take care about time management, this broad concept is discussed under the term of “polychronicity,” which is a result of demonstrated preferences of nurses that how much they are touchy to be engaged in a series of tasks or relevant events simultaneously and have trust that they are doing the best (Weintraub et al., 2019, 2020). Expectations of hospitals are high for nurses to perform multiple tasks simultaneously and take care of patients who show excellent services and their performance (Asghar et al., 2020).

For decades, researchers enlightened nurses who got pressurized to multitasks at work (Mittal and Bienstock, 2019) and home (Ophir et al., 2009); this trend is followed primarily in this complex world. This concept is more elaborated by Wei et al. (2018) that lack of sustained efforts of nurses can perpetuate errors and the loss of value of work completed. Kubicek and Tement (2016) have been suggested that multitasking is intensely related to WFC and FWC in both domains. Subsequently, evaluations were done on how non-work in which family and home lie as stressors created workplace interference. On the contrary, work stressors created interference with non-work WFC (Sanz-Vergel et al., 2015; Weintraub et al., 2019). According to prior literature, it is extended to infer that the conservation of resources theory provided a framework behind the linkage of PC and WFC (Alvaro et al., 2010; Hobfoll, 2011).

**Figure 1:**
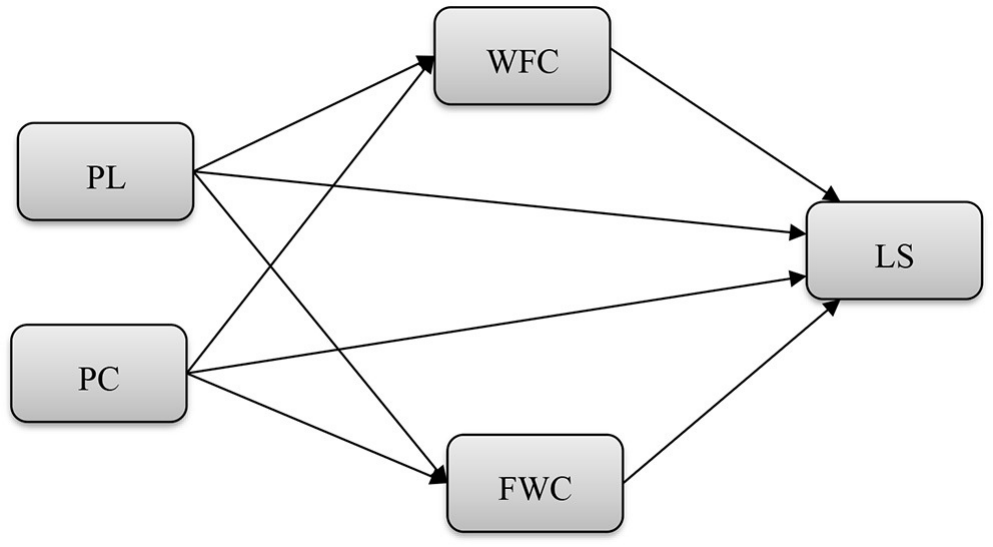
Study framework. PL, paternalistic leadership; PC, polychronicity; WFC, work-family conflict; FWC, family-work conflict; LS, life satisfaction.

Several researchers (Ugurluoglu et al., 2018; Weintraub et al., 2019) keenly studied a preference for PC, where people are involved in multiple roles and feel less overloaded instead of more monochronic direction. According to the study (Schieman and Young, 2010), work-family interference accelerated with the higher multitasking, particularly among those employees capable of controlling schedules according to the nature of their jobs. Consequently, people working on more than one task also possess an extraordinary sense of control over their lives and the time to manage tasks (Mittal and Bienstock, 2020). By extracting the relevant studies, it is proposed in this study that if nurses are polychronic, they can handle WFC and FWC resulting from which they can feel highly satisfied with their lives (see Figure 1).

*P6. PC is positively linked with LS*.
*P7. PC is negatively linked with WFC*.
*P8. PC is negatively linked with FWC*.
*P9. WFC mediates the relationship of PC with LS*.
*P10. FWC mediates the relationship of PC with LS*.

## Materials and Methods

### Sample and Data Procedure

In this study, we used a cross-sectional research design. This research design is more convenient and cost-effective for researchers, as data can be collected in a single time frame. Moreover, this design is beneficial and supportive for hypothesized model based on a theory (Teeroovengadum and Nunkoo, 2018). The convenience sampling technique was used to collect data from nurses of 16 public sector hospitals situated in the major cities of central Punjab, Pakistan. The reason for choosing the central Punjab area is that this is closely populated, comprising almost 41.6% (Pakistan Bureau of Statistics, 2021) of the total population of the Punjab province. The convenience sampling method was used because the nurses were overburdened with COVID patients and were not immediately available. So, we used our resources to approach them, disclosed the purpose of the study, and requested them to spare some time to fill the questionnaire. All the selected hospitals have different wards, but our focus was only on emergency and medical wards, where nurses performed multiple tasks at a time. Before distributing the questionnaires, we took prior approval from the concerned department to clarify the aim of the entire study and scope. The sample consisted of 350 questionnaires, 280 were filled and collected from employees, and 226 questionnaires were fully completed, and the response rate was almost 64.57% that is highly acceptable (Hair et al., 1998). The demographics of the respondents are presented in Table 1. In addition, the questionnaires did not require the name or signature of respondents to maintain full confidentiality and privacy.

**Table 1:** Demographic results.

**Demographics variables**	**Frequency**	**Percent**
**Gender**		
Female	226	100.00
**Age**		
18–25	14	6.19
26–30	34	15.04
31–35	52	23.00
36–40	54	23.89
41–above	72	31.85
**Marital status**		
Married	127	56.19
Unmarried	47	32.74
Divorced	52	23.00
**Position**		
Permanent	142	62.83
Contractual	84	37.16
**Parents to take care**		
Yes	162	71.68
No	64	28.31
**Job experience (in years)**		
1–5	18	7.90
6–10	42	18.58
11-20	72	31.85
20–above	94	41.59
**Number of children**		
No children	9	3.98
All children preschool age	77	34.07
Preschool and school-age	60	26.54
All school age	47	20.79
School age and college age	20	8.84
College age	13	5.75

### Measurement

#### Paternalistic Leadership

PL was measured as suggested by Aycan et al. (2013) using the 10-item short version of the paternalistic leadership questionnaire (PLQ) and a 6-point Likert scale (1 = strongly disagree; 6 = strongly agree). A sample item consists of “My Leader behaves like a senior family member toward his/her employees.”

### Polychronicity

Five items of the scale were adopted to measure the level of PC (Bluedorn et al., 1999; Lindquist and Kaufman-Scarborough, 2007). The question item consists of “I like to juggle several activities at the same time.” This study was used as a 5-point Likert scale for the response rates (5 = strongly agree; 1 = strongly disagree).

#### Work-Family Conflict

The WFC and FWC were measured using four items for WFC and using four items for FWC with a total eight items and a 5-point Likert scale (1 = strongly disagree; 5 = strongly agree) as suggested by Grzywacz and Bass (2003) and Grzywacz et al. (2002), respectively. A sample item consists of “My job reduces the effort I can give to home activities?” From work–family conflict and family–work conflict, the sample item consists of “Responsibilities at home reduce the effort I can devote to my job?”

#### Life Satisfaction

The LS scale was established by Diener et al. (1985) using a five-item short version of LS and a 5-point Likert scale (1 = strongly disagree; 5 = strongly agree). A sample item consists of “In most ways, my life is close to my ideal.”

The descriptive statistics of sample means, standard deviations, and Pearson's correlation (two-tailed) among this study constructs are shown in Table 2. None of the correlations was above 0.70, showing no multicollinearity issues in this study.

**Table 2:** Means, standard deviation, and correlations.

**Variables**	**1**	**2**	**3**	**4**	**5**
1. Life satisfaction	1				
2. Family–work conflict	−0.264^**^	1			
3. Work–family conflict	−0.156^*^	0.409^**^	1		
4. Polychronicity	0.421^**^	−0.533^**^	−0.171^*^	1	
5. Paternalistic leadership	0.232^**^	−0.212^*^	−0.342^**^	0.321^**^	1
Mean	3.171	2.121	1.321	3.212	3.432
Standard deviation (SD)	1.143	1.324	1.223	0.943	0.543

* *Indicates that the significant levels at P < 0.05 (two-tailed test)*.

** *Indicates that the significant levels at P < 0.01 (two-tailed test)*.

## Analysis and Results

The complex relationships among constructs required more comprehensive data analysis methods (Hair et al., 2011). The Smart PLS-SEM was used in current research, and “PLS-SEM” was employed to assess all study variables (Ali et al., 2018). The PLS-SEM can be used for both models, reflective and formative (Hair et al., 2019), which test the comparative complex model (Hair et al., 2011). Here, we chose the smart-PLS-SEM approach due to some reason (i.e., small sample size and complex study model) (Lowry and Gaskin, 2014; Schlittgen et al., 2016). There is no data assumption in the Smart-PLS method, and generally, there is no statistical issue in a small sample size (Hair et al., 2019).

### Measurement Model

This study analyzed the variance-based method by using PLS-SEM (Ringle et al., 2015). First, the measurement model was assessed, and the second structural model (Lowry and Gaskin, 2014). The assessment of the measurement model observed the relationships among all indicators and the constructs.

PL, paternalistic leadership; PC, polychronicity; WFC, work-family conflict, FWC, family-work conflict; LS, life satisfaction; Alpha, Cronbach's α; rho_A, reliability indices for each construct; CR, composite-reliability score; AVE, average-variance-extracted.

The validity and reliability of the measurement model were determined using the outer loading of indicators, Cronbach's α (Hair et al., 2016), composite-reliability score (CR), and average-variance-extracted values, as shown in Table 3 (Ringle et al., 2015; Richter et al., 2016). Moreover, validity was measured through two dimensions; the score of AVE and outer loading of indicators were used for the discriminant validity/convergent validity, which was deliberated with cross-loading (Hair et al., 2016). The outer-loading value was > 0.70 threshold value (Chin, 2010), and the AVE values and CR scores were above the cut-off points of 0.50 and 0.70 (Hair et al., 2016), indicating that the measurement model was internal reliability (Richter et al., 2016).

**Table 3:** Factor loading, Cronbach's α, composite-reliability score, and average-variance-extracted.

**Variables**	**Items**	**Factor loadings**	**Alpha**	**rho__**A**_**	**CR**	**AVE**
Polychronicity	PC1	0.771	0.896	0.929	0.925	0.614
	PC2	0.743				
	PC3	0.852				
	PC4	0.831				
	PC5	0.633				
Paternalistic leadership	PL1	0.865	0.963	0.968	0.898	0.649
	PL2	0.857				
	PL3	0.875				
	PL4	0.895				
	PL5	0.875				
	PL6	0.877				
	PL7	0.906				
	PL8	0.886				
	PL9	0.838				
	PL10	0.776				
Work-family conflict	WFC1	0.738	0.835	0.865	0.890	0.673
	WFC2	0.834				
	WFC3	0.922				
	WFC4	0.836				
Family-work conflict	FWC1	0.955	0.935	0.947	0.954	0.537
	FWC2	0.884				
	FWC3	0.923				
	FWC4	0.895				
Life satisfaction	LS1	0.853	0.862	0.916	0.902	0.698
	LS2	0.843				
	LS3	0.930				
	LS4	0.70				
	LS5	–				

The composite results of each construct have been measured by averaging the value across indicators indicating those variables. At the first level of the reflective measurement model, the alpha value of all the constructs is >0.70. The measurement model (rho_A) values are very close to Cronbach's α value, with the same threshold values applying to them. At the same time, reliability and consistency of all the indicators were assessed through outer loadings. Data were measured using outer loadings instead of convergent validity, and CR score, AVE value, and discriminant validity were assessed using cross-loadings, criteria, and the Heterotrait-Monotrait ratio (Hair et al., 2016). All the coefficients of alpha, CR, AVE, and rho–_A_ values were above the threshold limit (Hair et al., 2016), which shows the reliability and validity of reflective measurement models.

The convergent validity was measured by investigating the AVE value, which provides the total variance that a construct attained from its items with the total variance due to the dimension error (Richter et al., 2015). In Table 3, the AVE value >0.50 which shows the validity of the model and one item deleted from LS due to less than the cutoff point (Ringle et al., 2015); these findings recommend that the convergent validity of the measurement model is highly acceptable (Hair et al., 2016). The predictable methodology accompanies convergent validity and discriminant validity (Ringle et al., 2015).

### Structural Model

This study followed the suggestion of Hair et al. (2016) to confirm the structural model. The values of VIF were less than the threshold limit of 5–10. As a result, the structural model had no problems with multicollinearity among all predictors. They also suggested that multicollinearity did not make the structural model results pretentious. The structural model assessment was considered through the coefficient of determination *R*^2^ values for all the endogenous variables. The variance explained that *R*^2^ values of constructs (WFC = 0.401, FWC = 0.373, and LS = 0.592) are greater than a threshold limit as suggested by Ringle et al. (2015) and Lowry and Gaskin (2014), which reinforced the structural models in sample analytical power (Schlittgen et al., 2016). This study has measured the discriminant validity with two approaches: (a) discriminant validity measured by using for correlation between constructs and (b) cross-loadings (Zaiţ and Bertea, 2011). The predictive relevance (*Q*^2^) values are intended by the blindfolding method. This approach is used for path modeling; the cross-validated redundancy (*Q*^2^) value is >0, but this study recommends predictive relevance. The predictive relevance (*Q*^2^) values of all endogenous variables are shown in Table 4.

**Table 4:** Coefficients of determination and predictive relevance.

**Endogenous variables**	***R*^**2**^**	**Threshold value**	***Q*^**2**^**	**Threshold**
WFC	0.401	Moderate	0.284	> 0
FWC	0.373	Moderate	0.213	> 0
LS	0.592	Substantial	0.321	> 0

*R^2^, coefficients of determination; Q^2^, predictive relevance; WFC, work–family conflict; FWC, family–work conflict; LS, life satisfaction*.

A bootstrapping method used in this study (5,000 bootstrap samples; with no changes the sign) to create *t*-values with the level of significance, as well as *P*-values 95% CI in percentile, and bias-corrected was shown to assess hypothesis relationship among the variables in the study model (Hair et al., 2016). Table 5 shows that the PL (*β* = 0.149, *P* < 0.05) has a significant relationship with LS, PL (*β* = −0.161, *P* < 0.05), and WFC. Moreover, PL (*β* = −*0.106, P* < 0.05) has a significant relationship with FWC. The results of this study show that WFC and FWC partially mediate the relationship between PL and LS. WFC shows the partially mediating role between PL and LS (43%), and FWC partially mediates the association between PL and LS (58%). However, PC (*β* = 0.173, *P* <0.05) has a significant association with LS. P7: PC has a negative relationship with WFC (*β* = −0.221, *P* > 0.05) and PC is not associated with FWC. In addition, the WFC shows the partially mediating role (50%) and the association between PC and the LS of nurses. The FWC has no mediation effect between PC and LS. The effect size *f* ^2^ is represented to the small effects (0.02), medium effects (0.15), and the large effects (0.35), respectively (Cohen, 1992). This study measures the mediation analysis through the variance accounted (VAF) method; this method is most authentic comparatively with the Sobel test (Asghar et al., 2020). The VAF method is given as follows to compute the mediation analysis. The value of VAF > 80% shows the full mediation, and the VAF value <80% confirmations of partial mediation, and if the VAF value <20% that there is no mediation exists (Nitzl et al., 2016; Hair et al., 2019).

**Table 5:** Paths analysis.

**Hypothesis**	***β***	***t***	***f^**2**^***	***P***	**95% CI**	**Status**
PL → LS	0.149	13.14	0.027	0.001	(0.033, 0.273)	*(P1)* Supported
PL → WFC	−0.161	3.627	0.026	0.010	(0.020, 0.294)	*(P2)* Supported
PL → FWC	−0.106	2.132	0.014	0.021	(0.050, 0.295)	*(P3)* Supported
PC → LS	0.173	3.927	0.037	0.012	(0.155, 0.209)	*(P6)* Supported
PC → WFC	−0.221	2.210	0.102	0.001	(0.090, 0.344)	*(P7)* supported
PC → FWC	−0.102	0.970	0.006	0.330	(−0.030, 0.247)	*(P8)* ns
**Paths**	**IE**	**T**	**VAF**		M**ediation result**	–
PL→ WFC→ LS	0.113	0.262	43%		Partial mediation	*(P4)* Supported
PL→ FWC→ LS	0.088	0.249	58%		Partial mediation	*(P5)* Supported
PC→ WFC→ LS	0.141	0.042	50%		Partial mediation	*(P9)* Supported
PC→ FWC→ LS	0.054	0.276	19%		No mediation	*(P10)* ns

*β, coefficients; f^2^, effect size; P, significance level; 95% CI, confidence interval; IE, indirect effect; TE, total effect; VAF, variance accounted for; ns, not supported; PL, paternalistic leadership; PC, polychronicity; WFC, work-family conflict; FWC, family-work conflict; LS, life satisfaction*.

## Discussion

This study examined the effect of PL and PC on LS. Moreover, this study explored the mediating role of WFC and FWC among PL, PC, and LS. The results revealed that PL behavior positively affects LS of employees, whereas PL has a negative relationship with WFC and FWC. Moreover, WFC and FWC play a mediating role between PL and LS. The findings of this study show that there is a positive relationship between PC and LS. Also, PC has a negative relationship with WFC and FWC.

WFC and FWC partially mediate the association between PL and LS. According to previous studies, some leaders served as a role model for their followers, and kind-hearted leaders showed good-quality interpersonal relationships and support their followers (Mittal and Bienstock, 2019, 2020). Therefore, hypotheses P1–P7 and P9 are accepted, whereas hypotheses P8 and P10 are rejected.

In the healthcare sector, PL behavior practices need to be exercised to integrate work, leading to avoiding confusion positively. Basically, a leader is a person who plays a fundamental role to build and strengthen the quality of discipline and support their employees to accomplish the organizational objective (Hartley et al., 2008; Mauno et al., 2015). PL behavior showed concerns toward subordinates LS; it is also interconnected deeply with the well-being of employees (Chen, 2017; Yang and Fry, 2018). PL style of leadership played a vital role in the LS of nurses, which enhances performance (Wang et al., 2020). According to research from Brazil, employees who work under paternalistic leaders have faced less WFC (Cacique et al., 2019). As a result, paternalistic leaders promote an environment where employees feel comfortable and satisfied while performing their duties (Mittal and Bienstock, 2020).

In this study, we also proposed that polychronic employees are more satisfied with their lives than others, whereas WFC and FWC negatively affect their LS. Moreover, WFC and FWC play as a mediating role between PC of nurses and their LS. The findings of this study reveal that there is a positive relationship between PC and LS. WFC partially mediates the relationship between PC and LS, whereas FWC has no mediation effect. These findings enhance the interest of scholars while providing important considerations and results of our study in which WFC contradicts the previous studies because of culture differences and population (Lau et al., 2020; Mittal and Bienstock, 2020). Polychronic nurses, in particular, do their work effectively and efficiently, allowing them to be satisfied with their lives and the demands of work and family (Sehrish and Zubair, 2020). Nursing staff perform several tasks simultaneously in hospitals and are engaged in multiple activities (Lee-Peng et al., 2016), enhancing their polychronic behavior (Hertenstein et al., 2019). Based on COR theory, it is also indicated that polychronic employees have the capability to cope with several roles and responsibilities at a time (Hobfoll, 2011).

### Implications and Limitations

This study provides numerous theoretical and practical implications for hospitals, administrators, and policymakers. Theoretically, we tried to expand the COR theory by incorporating the PL, PC, and LS in one model. In addition, the mediation role of WFC and FWC was also integrated with the proposed model. Thus, this is one of the pioneer studies exploring the effects of PL, PC on LS through WFC and FWC in one model, specifically in the healthcare sector.

Today, retaining employees is a perilous issue in every organization, especially in hospital sectors, where nurses work in stressful working environments and need support from the leader. PL behavior can increase LS, commitment, and job performance of employees to attain organizational goals. This study provides some managerial suggestions to the administration of hospitals and policymakers regarding organizational context where factors are involved in increasing the PC that enhances the LS of nurses. Furthermore, the leaders should train their employees to cope with WFC and FWC that foster satisfaction of employees toward their life. The medical superintendent and administration must conduct the education and training sessions for female staff which explain how to handle the multiple tasks. Finally, hospital administration must create a sympathetic and healthy work environment that boosts the positive energy of employees to cope with problematic issues productively.

This research has some also limitations. The study design on the cross-sectional method that limits assumptions about connection and future research on this topic might use a longitudinal study design to observe the associations of given variables. It is noticed that the results of this study are partial in the research context and sample size. Further investigation is required in this research model. The generalizability of results is indispensable to be verified by different cultural perspectives. Moreover, the study model should be tested with other variables. Subsequently, data collection can be conducted in hospitals from different provinces of Pakistan. Finally, this research was conducted on just nurses working in the hospital environment and so future studies should examine the survey on other target populations.

## Conclusion

This study has strived to fill a gap in WFC and FWC literature by linking the PL style of the supervisor to the work and home conflicts of the followers. This study also establishes PC as an important predictor of the work and home conflicts and LS of employees. This study links PL behavior, PC, WFC, FWC, and LS in one model. The results foster the significance of this study because PL and PC are used as predictors simultaneously, meaning that if someone has the skill to work more and his/her leader supports him/her, he/she will be more satisfied in his/her life. Researchers in prior studies argue that leadership practices would affect job satisfaction, work-family balance, and overall well-being.

## Data Availability Statement

The raw data supporting the conclusions of this article will be made available by the authors, without undue reservation.

## Ethics Statement

The studies involving human participants were reviewed and approved by the Ethics Committee of Yanshan University, China. The participants provided their written informed consent to participate in this study.

## Author Contributions

NG and MA considered and designed the concept and wrote the paper and were involved in writing—original draft preparation and contributed to the data collection. NG and MAR were involved in writing—review and editing and performed the literature review and reviewed the work to improve the outcomes. RS and YL helped to provide technical support to collect the data. MA and ZS contributed to analysis tools. ZS has supervised and funded RS for this work. MAR and YL help us during revision (writing—review and editing and reviewed the work to improve the outcomes). All authors have read and agreed to the published version of the manuscript.

## Conflict of Interest

The authors declare that the research was conducted in the absence of any commercial or financial relationships that could be construed as a potential conflict of interest.

## Publisher's Note

All claims expressed in this article are solely those of the authors and do not necessarily represent those of their affiliated organizations, or those of the publisher, the editors and the reviewers. Any product that may be evaluated in this article, or claim that may be made by its manufacturer, is not guaranteed or endorsed by the publisher.
